# Vestibular rehabilitation in hereditary spastic paraplegia: a randomized pilot study

**DOI:** 10.1055/s-0046-1827050

**Published:** 2026-08-31

**Authors:** Geslaine Janaina Bueno dos Santos, Maria Izabel Rodrigues Severiano, Flávio Magno Gonçalves, Cristiano Miranda de Araujo, Bianca Simone Zeigelboim, Hélio Afonso Ghizoni Teive

**Affiliations:** 1 Universidade Federal do Paraná, Programa de Pós-Graduação em Medicina Interna e Ciências da Saúde, Curitiba PR, Brazil.; 2 Instituto Federal do Paraná, Departamento de Reabilitação, Curitiba PR, Brazil.; 3 Universidade Tuiuti do Paraná, Programa de Pós-Graduação em Saúde da Comunicação Humana, Departamento de Otoneurologia, Curitiba PR, Brazil.

**Keywords:** Rehabilitation, Virtual Reality, Spastic Paraplegia, Hereditary, Postural Balance

## Abstract

**Background:**

Hereditary spastic paraplegia (HSP) is a neurodegenerative disorder characterized by spasticity, lower limb weakness, impaired balance, and increased risk of falls.

**Objective:**

To evaluate the effect of vestibular rehabilitation associated with virtual reality (VRi) on functional balance in patients with HSP.

**Methods:**

A randomized pilot clinical trial was conducted with 16 patients who were diagnosed with HSP and divided into two groups: GI (balance games) and GII (balance + strength games). Interventions were performed via the Wii console and the Wii Balance Board platform (Nintendo Co, Ltd.). The Berg balance scale (BBS) and physiological profile assessment (PPA) were administered at T0 (prerehabilitation), T1 (after 10 sessions), and T2 (after 20 sessions). The Friedman test and Wilcoxon test were used to analyze temporal changes, the Mann–Whitney test was used to compare the groups.

**Results:**

All patients reported imbalance and muscle fatigue, whereas heaviness in the lower limbs and weakness were more common in GII. The visual contrast domain showed a significant change from T0 to T2, with scores increasing by a median of 2.0 (18.5–20.5) units.

**Conclusion:**

The use of VRi showed potential as a therapeutic adjunct in HSP rehabilitation, with improvements in balance and fall risk. However, larger studies are needed to corroborate these results.

**Clinical trial registration:**

ReBEC (RBR-3JMX67, 01/29/2020). https://ensaiosclinicos.gov.br/rg/RBR-3jmx67 .

## INTRODUCTION

Hereditary spastic paraplegia (HSP) comprises a heterogeneous group of rare, hereditary neurodegenerative disorders that affect the long axonal fibers of the corticospinal tracts in the spinal cord. 1 2 The corticospinal tract is responsible for transmitting motor signals from the cerebral cortex to the spinal cord and, subsequently, to the effector muscles, thereby controlling limb movements. 3

In HSP, the axons of motor neurons that transmit nerve impulses to the lower limbs (LL) are particularly vulnerable to degeneration. This retrograde process results in progressive weakness, spasticity, and difficulties in performing activities such as ambulation. 3 4 5 6 7

The disease originates from mutations in genes responsible for encoding crucial proteins that maintain the integrity of corticospinal tract neurons. 8 It can be classified as autosomal dominant (AD), autosomal recessive (AR), X-linked, or mitochondrial; 4 5 6 9 and further categorized as simple or pure (HSP-S) and complicated, complex, or plus (HSP-C). In the pure form, patients present a family history and a clinical picture predominantly characterized by progressive spastic paraplegia, gait disturbance, hyperreflexia, urinary incontinence, deep sensory abnormalities, and increased muscle tone in the LL. 6 10 11 12

In addition to the characteristics observed in the pure form, the complex form includes other manifestations, such as intellectual disability, peripheral neuropathy, seizures, visual impairments, cognitive deficits, parkinsonism, and cerebellar ataxia. 9 10 13 14 Although disease progression is generally slow, it can vary significantly among patients with the same genetic pattern, reflecting biological variability and the unpredictable nature of the disease. 10

Pure forms of HSP are generally transmitted in an AD manner (AD-HSPs), whereas complex forms often present additional symptoms and are more frequently associated with AR inheritance (AR-HSPs). The AD-HSP form accounts for approximately 80% of cases in Europe and North America. In Brazil, AR inheritance of HSP is as prevalent as the AD form because of the high incidence of consanguineous marriages in the population. 15 16

The estimated prevalence of HSP ranges from 1.3 to 9.6 per 100,000 individuals, depending on the geographic region. 17 The disease, is classified on the basis of phenotype, inheritance pattern, and mutated gene. 4 More than 80 genetic subtypes have been identified, covering all inheritance patterns, with onset ages ranging from childhood to 80-years-old. 18 19 In pure forms, the average late-onset age is approximately 40 years, whereas complex forms exhibit even greater variability. 19

Owing to weakness and spasticity in the LL, postural balance may be compromised. Balance is essential for maintaining posture and is sustained by the visual, vestibular, and proprioceptive systems. Accurate diagnosis and appropriate rehabilitation are crucial for preventing imbalance, falls, and other symptoms, such as dizziness and a sensation of instability. 19 20

Vestibular rehabilitation (VR) with virtual reality (VRi) has emerged as an important health-promoting strategy, leveraging the neuroplasticity of the central nervous system (CNS) to improve patients' functionality and safety. 20 21 This approach aims to enhance postural control through repetitive and specific physical exercises, stimulating the CNS to process and respond appropriately to sensory information. Key mechanisms include the vestibulo-ocular reflex (VOR), which stabilizes vision during head movements, and the vestibulospinal reflex (VSR), which maintains body stability. 21

The use of VRi has proven to be an effective therapeutic tool for patients with neurological disorders, promoting vestibulo-visual integration and improving both static and dynamic postural stability. Compared with traditional training, VRi offers significant advantages by stimulating the vestibular system and supporting CNS neuroplasticity processes, modifying its properties in response to environmental stimuli. 16 21 22 23 24

Furthermore, VRi platforms provide immersive experience in simulated environments that enhances spatial perception and triggers reflex responses related to existing symptoms. These platforms have been effective in treating individuals with neurodegenerative diseases. 16 21

An imbalance and fear of falling can impair daily activities, particularly those involving rapid head movements or trunk flexion, leading to falls and functional limitations. 21 25 These difficulties directly impact patients' quality of life (QoL), compromising their ability to perform daily tasks safely and independently. 16

The objective of the present study was to evaluate the effectiveness of VRi in improving balance and reducing the risk of falls in HSP patients.

## METHODS

The present study followed the checklist of the Consolidated Standards of Reporting Trials (CONSORT) guidelines. 26

### Study design

The present was a randomized pilot clinical trial with adult patients diagnosed with HSP from the Department of Neurology at the Hospital de Clínicas in Curitiba, PR, Brazil. The study was approved by the Research Ethics Committee of Sociedade Evangélica Beneficente (Beneficent Evangelical Society), under protocol number 3.580.973 (CAAE: 370.837.14.0.0000.0103) on September 19, 2019. The trial protocol was registered and approved on the ReBEC platform, number RBR-3JMX67, and was previously published. 21

### Participant selection

The present study included patients who were diagnosed with HSP and still underwent genetic testing for subtype determination. The diagnosis was based on individual and family history, clinical symptoms (LL spasticity), neurological examination findings, and laboratory and imaging assessments consistent with the disease patterns.

Participants were consecutively selected from February to September 2022, with recruitment ending upon completion of the rehabilitation period. All patients and/or their legal representatives provided written informed consent before participation.

The inclusion criteria were as follows: age ≥ 18 years (no upper age limit); men and women diagnosed with HSP; and ability to ambulate independently, with or without assistive devices. The exclusion criteria were otoneurological disorders that could interfere with vestibular examination results; inability to follow and understand simple verbal commands; severe visual impairment or other conditions that might prevent completion of the proposed procedures; and significant musculoskeletal conditions that could hinder assessment and VRi intervention.

All patients underwent an initial videonystagmography (VENG) otoneurological assessment to rule out middle ear impairment that could affect labyrinthine test results and to evaluate the presence of vestibular disorders. This assessment included vestibular function testing through various examinations related to labyrinthine and ocular function. The VENG examination comprised calibration of ocular movements, assessment of spontaneous and gaze-evoked nystagmus, oscillatory tracking tests, optokinetic nystagmus evaluation, and rotational and caloric tests. Vestibular system analysis was performed via a digital VENG system equipped with specialized software (VecWin2, Neurograff Eletromedicina Ltda.), a rotational chair (Ferrante, model COD 14200, rotation frequency: 0.01–0.5 Hz), a visual stimulator (EV VEC model, Neurograff Eletromedicina Ltda.), and an air caloric stimulator (NGR 05 model, Neurograff Eletromedicina Ltda.). 27

### Interventions

The VRi intervention was conducted via the Wii Fit Plus system, which includes the Wii console, Wii-Remote, and the Wii Balance Board (WBB; Nintendo Co., Ltd.). The WBB is equipped with pressure sensors that detect the player's position and movement, allowing gestures similar to real-life activities displayed on the screen. This device promotes both reactive and proactive postural control strategies by providing visual feedback and challenges that encourage performance improvement during gameplay.

The games were selected to induce balance challenges and postural adjustments through visual stimuli and techniques guiding vision and body orientation in space. The activities involved head, trunk, and lower limb movements, including flexion, extension, and pelvic rotation, and weight transfers forward and laterally. The participants received different interventions based on which group they were assigned to:

Group I: Patients underwent VR rehabilitation using only balance games: *Soccer Heading* , *Table Tilt* , *Tightrope Walk* , *Penguin Slide* , and *Perfect 10* .
Group II: Patients underwent virtual reality rehabilitation via both balance and muscle-strengthening games: *Single Leg Extension* , *Torso Twist* , *Sideways Leg Lift* , and *Single Leg Twist* .

Both groups utilized the Wii console, Wii-Remote, and Wii Balance Board (Nintendo Co., Ltd.). The games featured a scoring system to assess participants' performance during activities. These scores provided an objective analysis of progress, facilitating comparisons between groups. The final score was displayed at the end of each game.

**Figure 1:**
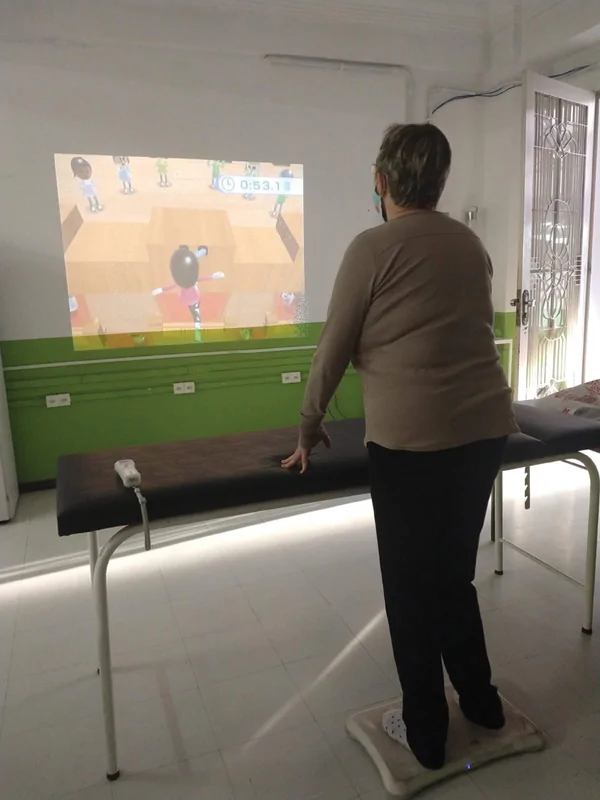
Patient performing balance training using a virtual reality system (Nintendo Wii and Wii Balance Board, Nintendo Co., Ltd.).

The participants were first introduced to the games and received instructions on the required movements. Each patient completed 20 VRi rehabilitation sessions, each lasting 50 minutes, twice a week. They completed the same assessment questionnaires at three time points: baseline (T0), after 10 sessions (T1), and after 20 sessions (T2). All the sessions were conducted in a controlled environment to minimize fall risk. The WBB was placed on the floor, with a treatment table positioned before it for support, if needed. To ensure participant safety, a researcher remained beside them throughout all the sessions. No dropouts occurred after the intervention began ( Figure 1 ).

### Outcomes

The Berg balance scale (BBS) and physiological profile assessment (PPA) instruments were applied before and after VRi rehabilitation in both groups at all three time points (T0, T1, and T2). General clinical and demographic data (age, sex, disease onset and duration) were recorded in a standardized electronic Excel spreadsheet (Microsoft Corp.). All data were collected by the outcome assessor, who remained blinded to group allocation throughout the study.

#### Berg balance scale (BBS)

The Brazilian version of the BBS was culturally adapted for the Brazilian population by Miyamoto et al. 28 and is used to identify risk factors for loss of independence and falls in various populations, especially older adults and patients with neurological disorders. Its specificity lies in its ability to objectively and comprehensively quantify an individual's capacity to perform a range of balance-related tasks, such as position changes, weight transfers, and static and dynamic balance maintenance.

The scale evaluates balance through 14 daily activities and is scored from 0 to 4, with a maximum possible score of 56 points, where higher scores indicate better balance. The final classification categorizes patients into three risk levels: low (good balance, low risk), moderate (moderate risk with some difficulty), and high (significant difficulty, high probability of falls), aiding in determining the need for interventions and support. 28 29

#### Physiological profile assessment (PPA)

The Physiological Profile Assessment (PPA), developed by Lord et al., 30 is a validated tool for assessing fall risk. It provides a detailed evaluation of sensorimotor abilities and is particularly effective in analyzing specific aspects, such as visual acuity (contrast sensitivity test), proprioception, lower limb muscle strength, reaction time, and postural balance, through static balance tests.

The obtained score classifies fall risk into four levels: < 0 (low risk), 0 to 1 (mild risk), 1 to 2 (moderate risk), and > 2 (high risk), allowing individual performance comparisons with normative values for the corresponding age group.

### Randomization

Before the intervention, patients were randomly assigned to two distinct groups. Randomization was performed via Random Allocation software (developed by M. Saghaei, Isfahan University of Medical Sciences, Iran), version 1.0.0, ensuring unbiased and impartial allocation of interventions.

### Blinding

Blinding was implemented at two independent levels. First, the outcome assessor remained unaware of group allocation throughout data collection. Second, the statistician responsible for data analysis was blinded until all analyses were completed. Allocation was determined by an independent researcher using a simple lottery system with opaque, sealed envelopes that were opened only after baseline assessment. Participant blinding was not feasible due to the interactive nature of the intervention, which required active engagement and, therefore, made it impossible to mask the assigned condition.

### Sample size

The current study was designed as a pilot randomized clinical trial; therefore, no formal sample size calculation was performed. The trial was registered with an operational recruitment estimate defined at the time of protocol submission. Because HSP is a rare condition, often associated with clinical and logistical challenges in recruitment, a reduced number of eligible participants was anticipated.

### Statistical analysis

Data normality was assessed via the Shapiro-Wilk test, whereas variance homogeneity was confirmed via Levene's test. To compare different time points (T0, T1, and T2) within each group (paired comparison), the Friedman test was applied. When statistically significant differences were observed, the Conover post hoc test was used for pairwise comparisons. The comparison between time points (T1 and T2) of the scores obtained in the different games was conducted via the Wilcoxon test.

To analyze differences between groups at different time points, two metrics were calculated:

- ∆1 (T1–T0): difference between the first and second time points.
- ∆2 (T2–T0): difference between the first and third time points.

These metrics (∆1 and ∆2) were compared between groups via the Mann–Whitney U test (unpaired comparisons at each time point) for the BBS and PPA values.

Finally, to ensure initial equivalence between groups, demographic and clinical variables were compared. Fisher's exact test was applied to categorical variables, such as age and disease duration, whereas the Mann–Whitney U test was used for numerical variables. All the statistical analyses were performed in the JASP 2023 (University of Amsterdam) software, version 0.17.2, with a significance level of 5%.

## RESULTS

**Figure 2:**
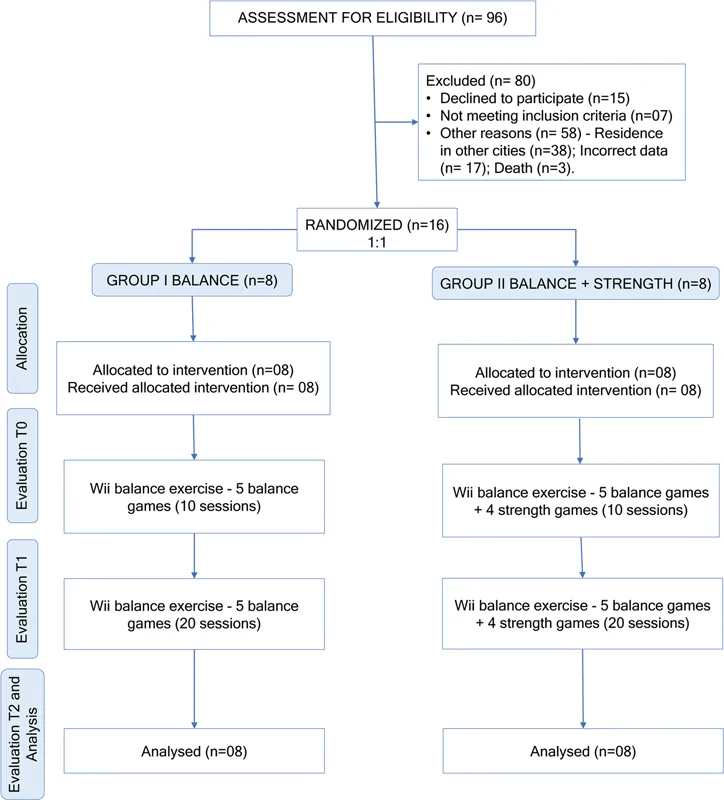
Consolidated Standards of Reporting Trials (CONSORT) flow diagram.

A total of 96 patients were assessed for eligibility, 16 of whom met the inclusion criteria and were enrolled in the study ( Figure 2 ). These 16 patients were evaluated.

The sample included a greater proportion of women (62.5%) than men (37.5%), in both groups ( *p* = 1.0). The disease duration ranged was 14 years for both groups ( *p* = 0.75). Vestibular examination revealed alterations in a significant number of patients. Bilateral deficient central vestibular dysfunction (GI: 50.0%; GII: 25.0%); Bilateral deficient peripheral vestibular dysfunction (GI: 0.0; GII: 37.5%), Left central irritative vestibular dysfunction (GI: 12.5%; GII: 12.5%) and normal exam (GI: 37.5%; GII: 25.0%; *p* = 0.28).

**Figure 3:**
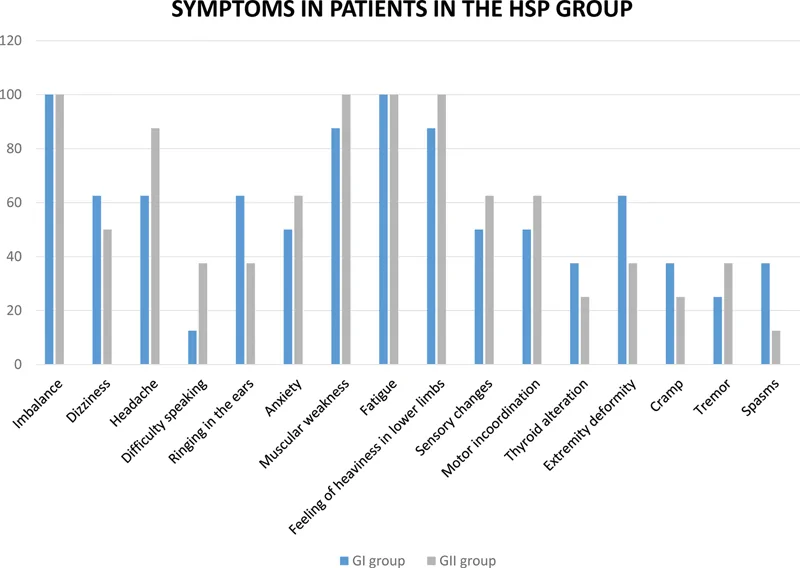
Symptoms in patients in the hereditary spastic paraplegia group.

The main complaints reported during the initial anamnesis were imbalance and muscle fatigue (100%) in both groups, a sensation of LL heaviness and muscle weakness (87.5%) in GI and (100%) in GII. In addition to gait difficulty due to lower limb spasticity, imbalance was the most prominent symptom observed and reported by patients ( Figure 3 ).

### Between-group comparison

No significant differences were observed between GI and GII in the BBS and PPA results. The same was true when comparing the scores obtained in the balance games between groups ( Table 1 ).

**Table 1:** Comparison between groups at each evaluation time point (∆1 and ∆2) for the BBS, PPA, and game scores

Domain	∆ GI	∆ GII	*p* -value*
	Median (IQR)	Median (IQR)	
∆ **BBS scores**			
BBS ∆1	1.00 (11.00)	0.00 (1.25)	0.746
BBS ∆2	1.52 (10.25)	3.00 (7.75)	0.874
∆ **PPA scores**			
PPA Z ∆1	-1.36 (1.52)	-0.425 (1.30)	0.574
PPA Z ∆2	-1.74 (2.24)	-0.83 (0.92)	0.442
**∆ Domains PPA**			
Sensitivity ∆1	1.50 (1.50)	0.50 (1.50)	0.384
Sensitivity ∆2	2.50 (4.00)	0.50 (2.25)	0.423
Proprioception ∆1	-0.70 (1.30)	-0.30 (1.15)	1.000
Proprioception ∆2	-0.80 (1.55)	-0.50 (2.25)	1.000
Muscle strength ∆1	3.00 (6.00)	2.00 (13.50)	0.832
Muscle strength ∆2	5.00 (11.00)	6.00 (10.50)	0.832
Reaction time ∆1	-27.30 (81.85)	-9.45 (27.12)	0.318
Reaction time ∆2	-23.55 (62.10)	-9.05 (75.82)	0.328
Balance test ∆1	-481.15 (732.68)	-7.73 (294.14)	0.505
Balance test ∆2	-477.65 (834.56)	-130.72 (457.26)	0.442
**Games**			
Soccer Heading	40.25 (45.87)	19.75 (54.50)	0.798
Table Tilt	14.25 (14.50)	15.75 (5.56)	0.752
Tightrope Walk	4.62 (6.87)	4.12 (7.50)	0.634
Penguin slide	6.37 (11.06)	7.12 (9.68)	0.753
Perfect 10	1.37 (2.18)	2.00 (1.18)	0.342

Abbreviations: BBS, Berg balance scale; PPA, physiological profile assessments; IQR, interquartile range.

Notes: *Mann–Whitney U test; ∆1, T1–T0; ∆T2, T2–T0. GI, balance games; GII, balance + strength games.

### Within-group comparison

When comparing different evaluation time points (T0, T1, and T2) for the overall Z score, no statistically significant differences were observed between time points. However, when only the visual contrast domain was considered, statistically significant differences were found between the scores at T0 and T2. Similarly, the BBS scores did not significantly differ ( *p* > 0.05), as presented in Table 2 .

**Table 2:** Comparative analysis of T0, T1, and T2, focusing on the overall score and PPA variables and the BBS, with the results presented separately for GI (balance) and GII (balance + strength)

	**GI (** ***n*** ** = 8)**				**GII (** ***n*** ** = 8)**			
	**T0**	**T1**	**T2**	***p*** -value*	**T0**	**T1**	**T2**	***p*** -value*
PPA score Z	3.89 (1.98) ^a^	2.35 (2.11) ^a^	1.47 (1.47) ^a^	0.077	3.40 (2.35) ^a^	1.65 (1.91) ^a^	1.39 (1.46) ^a^	0.067
Contrast sensitivity	18.50 (2.25) ^a^	19.50 (1.75) ^ab^	20.50 (4.25) ^b^	0.048*	19.50 (1.25)ª	20.00 (3.25)ª	19.50 (4.25)ª	0.113
Proprioception	1.50 (1.50)ª	1.00 (0.50)ª	0.90 (0.25)ª	0.331	2.10 (2.15)ª	1.40 (0.65)ª	1.00 (0.05)ª	0.446
Knee extension	26.00 (15.00)ª	24.00 (12.00)ª	34.00 (10.50)ª	0.085	21.00 (15.00)ª	25.00 (6.00)ª	33.00 (12.50)ª	0.317
Reaction time	301.00 (127.8)ª	271.45 (75.72)ª	255.10 (40.52)ª	0.093	270.20 (57.75)ª	279.50 (90.72)ª	273.95 (92.45)ª	0.140
Balance test	772.38 (839.90)ª	389.24 (326.65)ª	338.35 (266.44)ª	0.135	614.04 (634.50)ª	229.33 (634.71)ª	311.06 (253.52)ª	0.368
BBS score	42.00 (25.25) ^a^	44.50 (28.50) ^a^	47.50 (16.50) ^a^	0.733	41.50 (16.00) ^a^	44.00 (20.75) ^a^	47.00 (14.75) ^a^	0.163

Abbreviations: BBS, Berg Balance Scale; IQR, interquartile range; PPA, Physiological Profile Assessment; T0, baseline; T1, after 10 sessions; T2, after 20 sessions.

Notes: Values are presented as median (IQR). Friedman test ( *p* < 0.05). Different superscript letters (a, b) within the same row indicate statistically significant differences between time points in the post hoc analysis ( *p* < 0.05). Values sharing the same letter are not significantly different. GI, balance games; GII, balance + strength games.

When the scores of different games were compared, significant differences were observed in all balance-related games for both groups GI and GII and in most strength-based games for group GII. The comparisons of the game scores for both groups are shown in Table 3 .

**Table 3:** Comparison between T1 and T2 for balance- and strength-based exergames, separated by groups GI (balance) and GII (balance + strength)

**Games**	**GI (** ***n*** ** = 8)**			**GII (** ***n*** ** = 8)**		
	**T1**	**T2**	***p-*** **value** *	**T1**	**T2**	***p-*** **value** *
**Balance**						
TableTilt	50.00 (15.81)	63.00 (28.43)	0.014*	48.75 (12.50)	64.87 (19.62)	0.014*
Tightrope Walk	28.12 (10.87)	34.12 (5.56)	0.059*	23.87 (10.31)	28.00 (9.37)	0.036*
Penguin slide	51.62 (8.56)	58.00 (20.06)	0.023*	50.00 (10.81)	60.12 (18.06)	0.055*
Perfect 10	13.12 (8.43)	14.75 (8.06)	0.036*	13.12 (4.37)	14.87 (556)	0.014*
Soccer Heading	39.25 (19.25)	80.62 (69.50)	0.008*	36.75 (20.12)	52.37 (70.12)	0.008*
**Strength**						
Single leg extension	–	–	–	91.87 (7.06)	96.50 (3.93)	0.022*
Torso Twist	–	–	–	75.12 (27.00)	72.00 (20.93)	0.109
Sideways leg lift	–	–	–	90.87 (13.37)	97.50 (5.06)	0.022*
Single leg twist	–	–	–	84.87 (19.12)	91.50 (14.00)	0.008*

Abbreviation: IQR: interquartile range.

Notes: Values were presented as Median (IQR). *Wilcoxon signed-rank test ( *p* < 0.05). T1: after 10 sessions; T2: after 20 sessions; (-) group did not perform strength exercise.

## DISCUSSION

The use of VR has several benefits for patients with motor impairments. 21 22 23 31 32 33 However, no prior studies have explored its application in individuals with HSP. Thus, preliminary studies are needed to assess the feasibility, safety, and efficacy of this technology in this specific context. Accordingly, the present pilot study aimed to evaluate the impact of the VRi on balance and treatment efficacy in patients with HSP.

The results indicated a progressive improvement in balance and strength game scores, suggesting consistent performance enhancements over the sessions. Additionally, a significant improvement was observed in the PPA's visual contrast domain after the rehabilitation sessions, reinforcing the potential of VR as a complementary rehabilitation tool for these patients.

Patients with muscle weakness and imbalance are more prone to falls and have greater concerns related to fear of falling. 28 The relationship between balance and fall risk suggests that greater static and dynamic imbalances increase the likelihood of falls during ADLs. 29 This finding aligns with the present study, where improvements were observed in balance and strength game scores for both groups when T1 and T2 were compared. Thus, rehabilitation may play a role in enhancing and maintaining static and dynamic balance in patients with HSP, thereby reducing the risk/fear of falling.

Previous studies 29 have analyzed the five PPA domains and Z scores before and after intervention and failed to identify statistically significant differences in initial mean values between groups. However, significant results emerged after VRi game intervention in older adults without neurological impairment, corroborating our findings. In the present study, patients showed an improvement in visual contrast only in GI, when T0 and T2 were compared. This may be explained by the greater efficacy of the activities performed in promoting the integration of visual perception and motor control, which favor visuomotor coordination, stimulate neuroplasticity, and refine motor responses to visual stimuli. 34 35

Previous studies using the BBS have demonstrated statistically significant results in various neurodegenerative diseases, highlighting its applicability in assessing static and dynamic balance improvements. However, only one study has associated BBS with HSP, emphasizing compensatory actions between increased trunk movements and decreased balance capacity in affected individuals. 24 That study revealed that HSP patients with moderate trunk movements scored lower on the BBS and the Mini-Balance Evaluation Systems Test (Mini-BESTest) balance assessments than did those with normal movements. 24 Additionally, a study involving 13 patients with uncomplicated HSP evaluated the impact of a 6-week robotic-assisted gait training program. This protocol resulted in significant improvements in balance (assessed by the BBS), gait, and QoL, with effects maintained after 2 months. 36 However, in the present study, no statistically significant differences were observed in BBS scores. This result may be attributed to the small effect size, which is characteristic of pilot studies and may have limited the detection of significant differences. Thus, future studies with larger samples and greater statistical power are needed to evaluate this outcome more robustly.

The findings of the present study align with the literature, which highlights VR-based rehabilitation as a promising intervention for neurological disorders. 16 22 23 The use of VRi games has been shown to be effective in improving balance, reducing fall risk, and increasing functionality in various neurological conditions. 16 22 23 Older adults undergoing interventions with balance and muscle strength games have demonstrated improved reaction times and reduced body sway. 30 Additionally, games such as Soccer Heading and Ski Slalom have been associated with significant balance performance improvements in patients with SCAs. 22

In the current study, VRi games contributed to progressive improvements in balance and strength scores, demonstrating the feasibility of this approach for patients with HSP. The participants reported perceived benefits, such as increased confidence in daily activities and a reduced fear of falling. These results are consistent with previous studies associating VRi based VR with greater motivation and the creation of an immersive environment, which favors functional gains in patients. 20 21

Given the rarity of HSP and the clinical and logistical challenges involved in recruiting individuals with progressive neurodegenerative disorders, such as difficulty establishing contact, functional limitations, and limited availability for participation, the number of eligible participants was reduced. Consequently, the results of the present study should be interpreted with caution, particularly considering its pilot nature, the small sample size, and the potential methodological biases inherent to feasibility-oriented designs. Although the limited sample reduces the statistical power to detect differences between groups, especially for outcomes with small effect sizes, the improvement trends observed in some measures suggest a possible clinical benefit and reinforce the relevance of future studies with larger samples and adequate statistical power.

In conclusion, the present pilot study suggests that VRi based rehabilitation may be associated with improvements in balance, particularly in the visual contrast domain, in patients with HSP, when comparing T0 and T2, regardless of group allocation. Additionally, trends toward progressive improvement were observed in balance and strength game scores across sessions, indicating a possible positive effect of the intervention. Although no statistically significant differences were identified between groups, these findings support the feasibility and acceptability of VRi as a complementary and potentially motivating approach in the rehabilitation of patients with HSP. However, given the pilot design, small sample size, and use of nonparametric tests, the results should be interpreted with caution, highlighting the need for future studies, with greater statistical power and longitudinal follow-up, to confirm these findings.
